# Comparison of risk behaviors and socio-cultural profile of men who have sex with men survey respondents recruited via venues and the internet

**DOI:** 10.1186/1471-2458-10-232

**Published:** 2010-05-06

**Authors:** Hi Yi Tsui, Joseph TF Lau

**Affiliations:** 1 Centre for Health Behaviours Research, School of Public Health and Primary Care Faculty of Medicine, The Chinese University of Hong Kong, Hong Kong, Hong Kong

## Abstract

**Background:**

Increasingly more men who have sex with men (MSM) are using the internet to seek sex partners, and many HIV-related studies targeting MSM collect data from gay venues in order to inform the design of prevention programs. However, internet-based MSM may have different HIV risk behaviors and associated factors from those attending venues. This study examined differences in risk behaviors and socio-cultural profiles between MSM recruited from venues (e.g., gay bars/saunas) and from the internet respectively.

**Methods:**

An anonymous cross-sectional survey was conducted. A total of 566 Chinese MSM (340 recruited from gay-venues and 226 recruited from the internet) who self-reported having had anal or oral sex with another man in the last 12 months completed a structured questionnaire.

**Results:**

Internet-based MSM were more likely than venue-based MSM to have engaged in unprotected anal intercourse (53.3% *vs. *33.8%) or commercial sex (as clients: 12.8% *vs. *5.3%; as sex workers: 6.2% *vs. *1.5%), to have sought MSM partners from the internet (51.3% *vs*. 20.9%), and to have contracted sexually transmitted diseases (STD) in the last 12 months (4.4% *vs. *0.3%). On the other hand, internet-based MSM were less likely to have multiple sex partners (58.4% *vs. *75.6%) and to have used psychoactive substances (7.1% *vs. *15.6%) or drunk alcohol before sex (8.8% *vs. *16.2%). Moreover, internet-based MSM reported poor acceptance of their own sexual orientation, felt more discriminated against, and received less social support than venue-recruited MSM.

**Conclusions:**

Significant differences were observed between the two groups of MSM. Segmentation and targeted interventions are recommended when designing preventive interventions.

## Background

The prevalence of HIV among men who have sex with men (MSM) has been increasing in different parts of the world [1-5]. Although venue-based sampling has frequently been used in studies targeting MSM [6-9], the internet is becoming a potentially useful and cost-effective option [10-13]. Internet-recruited MSM, as compared to venue-based MSM, were in general more likely to report different socio-demographic profiles and higher levels of risk behaviors such as unprotected anal intercourse (UAI) [14,15], though mixed results have been reported [14-16]. Most studies investigating risk factors in association with UAI sampled MSM from gay-venues [e.g., 6,8,9] but it is unknown whether internet-based MSM have different risk factors. There is a dearth of data comparing risk factors among MSM recruited from different sampling spaces [11,12,14-16].

The HIV prevalence among MSM in Hong Kong, China in 2007 was around 4% [17], which is comparable to rates reported in some other Chinese cities [18]. A previous population-based study showed that around 2% of the general adult male population in Hong Kong self-reported having sex with men in the last 6 months and practicing risk behaviors such as UAI [19-21]. Moreover, a substantial proportion of MSM both in Hong Kong and in mainland China seek their male sex partners via gay venues or via the internet [20,22] and MSM in Hong Kong are interacting intensively with MSM in other Chinese cities [21,23].

The present study compared socio-cultural factors and levels of risk behaviors among Hong Kong MSM participants recruited via venue-based and internet-based sampling methods. The variables compared include socio-demographics, prevalence of self-reported sexually transmitted diseases (STD), risk behaviors, service utilization and socio-cultural characteristics. The associations between these factors and UAI were investigated separately for venue-based and internet-based participants and these 2 sets of factors were compared.

## Methods

### Study population and data collection

The study population comprised Chinese men aged between 18 and 60 years in Hong Kong who self-reported having engaged in anal or oral sex with men in the last 12 months. A total of 566 MSM completed an anonymous structured questionnaire - 340 were venue-based and 266 were internet-based. Venue-based MSM respondents were recruited via on-site convenience sampling from 4 gay bars, 2 gay saunas, and one beach frequently visited by local MSM. The same sampling method has been used in other similar studies [e.g., 9]. With verbal informed consent, face-to-face interviews were conducted by 3 well-trained peer interviewers between June and October, 2005. Each questionnaire took about 15 minutes to complete. An incentive of HK$50 (about 6.4US$) was offered to respondents for completion of the interview. The response rate, defined as the number of respondents completing the questionnaire divided by the number of eligible respondents invited to join the study, was approximately 64%.

During the same period, an identical anonymous self-administered online questionnaire (with the same briefing) was used to recruit participants from the internet. Peer workers of the project promoted the study and posted a URL link to the questionnaire on the discussion forums of local gay websites. These websites are popular and frequented by MSM in Hong Kong. The websites have chat-rooms, addresses of local gay venues, and partner-finding and other functions. Potential internet-based MSM respondents were asked to confirm eligibility before joining the study. Informed consent was implied by the return of completed questionnaires. No incentive was offered due to the lack of face-to-face contact and the anonymous nature of the study. Ethics approval was obtained from the Ethics Committee of the Chinese University of Hong Kong.

### Measurements

Information on respondents' age, education level, employment status (whether working full-time), and self-identified sexual orientation (homosexual, bisexual, heterosexual, or not certain) was collected. Respondents were asked whether they had been tested for HIV antibody or had received other types of HIV-related prevention services such as peer outreach education, condom and lubricant distribution, and educational messages on websites and magazines in the last 12 months. Three questions addressed HIV-related knowledge (see Table 1) and the number of items with appropriate responses was counted to create a binary variable (≤ 2 versus 3 appropriate answers). Three other questions (see Table 1) asked about participants' HIV-related perceptions, such as perceived susceptibility of acquiring HIV infection.

**Table 1:** Background characteristics of the respondents

	*All*	Group V	Group I	
	*(n = 566)*	(n = 340)	(n = 226)	Univariate OR
				
	*Col%*	Col%	Col%	*(Group I vs. Group V)*
Socio-demographics				
**Age groups**				
18-29	*70.0*	68.2	72.6	1.00
≥30	*30.0*	31.8	27.4	0.81
				
**Education level**				
≤ High school	*32.3*	37.4	24.8	1.00
≥College/university	*67.7*	62.6	75.2	1.81**
				
**Employment status**				
Employed full-time	*68.6*	79.4	52.2	1.00
Not employed full-time	*31.4*	20.6	47.8	3.53***
				
**Self-identified as exclusively homosexual**				
Yes	*81.1*	82.1	79.6	1.00
No (bisexual/not certain)	*18.9*	17.9	20.4	1.17
				
HIV-related prevention services (last 12 months)				
**Tested for HIV**				
No	*73.7*	71.8	76.5	1.00
Yes	*26.3*	28.2	23.5	0.78
				
**Received other HIV prevention services**				
No	*54.4*	54.1	54.9	1.00
Yes	*45.6*	45.9	45.1	0.97
				
HIV-related knowledge & perceptions				
**A healthy-looking HIV- infected person could transmit HIV to others#**				
Inappropriate answer	*20.8*	18.5	24.3	1.00
Appropriate answer (Yes)	*79.2*	81.5	75.7	0.71
				
**HIV virus could be transmitted via mouth-to-mouth kissing with an HIV-infected person#**				
Inappropriate answer	*64.7*	59.7	72.1	1.00
Appropriate answer (No)	*35.3*	40.3	27.9	0.57**
				
**HIV infection can be detected via blood test one week after the infection took place#**				
Inappropriate answer	*20.1*	20.9	19.0	1.00
Appropriate answer (No)	*79.9*	79.1	81.0	1.12
				
**Total number of appropriate responses to the above 3 items**				
≤ 2 appropriate answers	*75.6*	73.2	79.2	1.00
3 appropriate answers	*24.4*	26.8	20.8	0.72
				
**Perceived condom efficacy for HIV prevention**				
Quite high to low	*58.3*	63.8	50.0	1.00
Very high	*41.7*	36.2	50.0	1.76**
				
**Self-perceived susceptibility of HIV infection in the future**				
Little/very little	*66.6*	60.9	75.2	1.00
Moderate to very high	*33.4*	39.1	24.8	0.51***
				
**Fear of getting HIV via MSM sex behaviors**				
No	*35.5*	33.8	38.1	1.00
Yes/a little	*64.5*	66.2	61.9	0.83

Group V: Venue-recruited MSM; Group I: Internet-recruited MSM. **#**Response categories include "yes", "no", and "not certain".

*p < 0.05; **p < 0.01; ***p < 0.001.

Questions were also asked about participants' HIV-related behaviors in the last 12 months, including the number of MSM sex partners, having sex with different types of MSM partners, engagement in anal intercourse with MSM (and if so, whether condoms were used consistently during these sexual encounters), consumption of alcohol before having sex with MSM, use of psychoactive substances, and contraction of STD (Table 2).

**Table 2:** Sex partnerships, unprotected anal intercourse, substance use behaviors, and self-reported STD in the last 12 months

	*All*	Group V	Group I	
	*(n = 566)*	(n = 340)	(n = 226)	Univariate OR
				
	*Col%*	Col%	Col%	*(Group I vs. Group V)*
Number & type of MSM partner (last 12 months)				
				
**Number of MSM sex partners**				
One	*31.3*	24.4	41.6	1.00
More than 1	*68.7*	75.6	58.4	0.45***
				
**Having sex with MSM acquaintances recruited online**				
No	*67.0*	79.1	48.7	1.00
Yes	*33.0*	20.9	51.3	4.00***
**Having sex with male sex workers**				
No	*91.7*	94.7	87.2	1.00
Yes	*8.3*	5.3	12.8	2.63**
**Having provided commercial sex to males**				
No	*96.6*	98.5	93.8	1.00
Yes	*3.4*	1.5	6.2	4.43**
				
Anal intercourse with MSM (last 12 months)				
**Engaged in any MSM anal intercourse**				
No	*19.8*	20.0	19.5	1.00
Yes	*80.2*	80.0	80.5	1.03
				
*Among those who had engaged in any MSM anal intercourse (n = 454), * Unprotected anal intercourse (UAI) (last 12 months)				
**Engaged in any MSM UAI**				
No	58.4	66.2	46.7	1.00
Yes, UAI	41.6	33.8	53.3	2.23***
				
Substance use (last 12 months)				
**Used psychoactive substances**				
No	*87.8*	84.4	92.9	1.00
Yes	*12.2*	15.6	7.1	0.41**
**Drank alcohol before sex**				
No	*86.7*	83.8	91.2	1.00
Yes	*13.3*	16.2	8.8	0.50*
				
Self-reported STD (last 12 months)				
**Self-reported contraction of STD**				
No	*98.1*	99.7	95.6	1.00
Yes	*1.9*	0.3	4.4	15.69**
				

Group V: Venue-recruited MSM; Group I: Internet-recruited MSM.

*p < 0.05; **p < 0.01; ***p < 0.001.

Five items assessed participants' level of self-acceptance for their MSM sexual orientation. Three items assessed participants' perceived discrimination against MSM. Five other items assessed participants' perceived social support toward their MSM behaviors. These items are listed in Table 3. The total number of responses indicating non-acceptance of own sexual orientation (≤ 2 versus 3 to 5 non-acceptance responses), perceived discrimination (≤ 2 versus 3 perceived discrimination responses), and perceived social support (≤ 3 versus 4 to 5 responses indicating social support) were used to form 3 binary indicator variables.

**Table 3:** Perceived rejection, self non-acceptance and social support in relation to MSM behaviors

	*All*	Group V	Group I	
	*(n = 566)*	(n = 340)	(n = 226)	Univariate OR
				
	*Col%*	Col%	Col%	*(Group I vs. Group V)*
Self-acceptance of MSM sexual orientation				
**Felt uneasy about their sexual orientation**				
No	*64.5*	71.5	54.0	1.00
Yes	*35.5*	28.5	46.0	2.14***
**Felt ashamed of their sexual orientation**				
No	*78.4*	84.4	69.5	1.00
Yes	*21.6*	15.6	30.5	2.38***
**Afraid others knowing their sexual orientation**				
No	*36.0*	45.9	21.2	1.00
Yes	*64.0*	54.1	78.8	3.14***
**Fully accept their sexual orientation**				
Yes	*88.7*	92.0	83.6	1.00
No/a little	*11.3*	8.0	16.4	2.26**
**Would date/marry a woman to hide their sexual orientation**				
No	*75.1*	82.4	64.2	1.00
Yes(already did so/may be)	*24.9*	17.6	35.8	2.61***
**Number of items showing self non-acceptance of MSM behaviors (to the above 5 items)**				
0 to 2 non-acceptance responses	*74.4*	81.8	63.3	1.00
3 to 5 non-acceptance responses	*25.6*	18.2	36.7	2.60***
				
Perceived MSM-related discrimination				
**Perceived discrimination against MSM in Hong Kong**				
Very little/little	*37.5*	50.3	18.1	1.00
A fair amount/a great deal	*62.5*	49.7	81.9	4.57***
**Felt being discriminated because of their sexual orientation**				
No	*37.3*	51.8	15.5	1.00
Yes	*62.7*	48.2	84.5	5.86***
**Experienced pressure from family & friends due to their sexual orientation**				
No	*34.7*	40.1	26.5	1.00
Yes	*65.3*	59.9	73.5	1.85**
**Number of items showing perceived discrimination against MSM (to the above 3 items)**				
0 - 2 discrimination responses	*61.3*	75.3	40.3	1.00
3 discrimination responses	*38.7*	24.7	59.7	4.52***
				
Perceived social support				
**Family knowing their sexual orientation**				
A few or none	*78.4*	75.6	82.7	1.00
All or most of them	*21.6*	24.4	17.3	0.64*
**Family supporting their sexual orientation**				
No/unaware of one's sexual orientation	*83.7*	79.1	90.7	1.00
Yes	*16.3*	20.9	9.3	0.39***
**Best friends supporting their sexual orientation**				
No/unaware of one's sexual orientation	*36.0*	30.6	44.2	1.00
Yes	*64.0*	69.4	55.8	0.56**
**Having someone to talk to about their sexual orientation**				
No	*26.9*	17.4	41.2	1.00
Yes/no need	*73.1*	82.6	58.8	0.30***
**Number of friends having MSM behaviors**				
A few or none	*58.8*	46.6	77.0	1.00
All or most of them	*41.2*	53.4	23.0	0.26***
**Perceived extent of support received regarding MSM behaviors (to the above 5 items)**				
0 - 3 support responses	*83.9*	78.8	91.6	1.00
4 -5 support responses	*16.1*	21.2	8.4	0.34***

Group V: Venue-recruited MSM; Group I: Internet-recruited MSM.

*p < 0.05; **p < 0.01; ***p < 0.001.

### Statistical analysis

Univariate logistic regression analyses were performed to compare between-group differences (internet-recruited participants or *Group I *versus venue-recruited participants or *Group V*). As the independent variables listed in Tables 1, 2 and 3 were inter-correlated, a summary multivariate model was fitted to identify variables that were independently associated with the mode of recruitment. Variables showing significant univariate between-group differences were used as candidate variables in a stepwise multivariate logistic regression model discriminating Group I versus Group V respondents. Separate univariate and multivariate logistic regression analyses were conducted to identify factors in association with UAI in the two groups (Group I and Group V). All statistical analyses were performed using SPSS for Window 14.0 and a p-value < 0.05 was taken as statistically significant.

## Results

### Background characteristics

Results are summarized in Table 1. Compared to Group V respondents, Group I respondents were statistically more likely to have attended college or university (75.2% versus 62.6%), to not be working full-time (47.8% versus 20.6%), and to perceive condom use as being efficacious for HIV prevention (50.0% versus 36.2%). Group I respondents were less likely than Group V to have provided an appropriate response to the item concerning whether mouth-to-mouth kissing with an HIV-infected person could transmit HIV (27.9% versus 40.3%), and to perceive being moderately or highly susceptible to contracting HIV (24.8% versus 39.1%).

### Risk behaviors and self-reported STD in the last 12 months

Group I respondents were more likely than Group V respondents to report certain risk behaviors, including having had sex with male sex workers (12.8% versus 5.3%), recruiting MSM sex partners from the internet (51.3% versus 20.9%), providing commercial sex services to other MSM (6.2% versus 1.5%), being inconsistent condom users during anal sex (53.3% versus 33.8%), and having contracted STD in the last 12 months (4.4% versus 0.3%; Table 2). The reverse was true for having had multiple MSM sex partners in the last 12 months (58.4% versus 75.6%), and use of psychoactive substances or alcohol prior to having sex in the last 12 months (psychoactive substances: 7.1% versus 15.6%; alcohol: 8.8% versus 16.2%; Table 2).

### Socio-cultural variables

Group I respondents were more likely than Group V participants to have given "non-acceptance" responses to the 5 individual items assessing acceptance of their sexual orientation (univariate OR ranged from 2.14 to 3.14, p < 0.01) and to have provided ≥ 3 responses (out of 5) indicating non-acceptance of their sexual orientation (36.7% versus 18.2%, univariate OR = 2.60, p < 0.001; Table 3).

Of all respondents, respectively 62.5%, 62.7% and 65.3% reported perceiving a fair amount to a great deal of discrimination against MSM in Hong Kong, feeling discriminated against, or experiencing pressure from family/friends due to their sexual orientation (Table 3). Group I respondents were more likely than Group V respondents to have provided responses indicating perceived discrimination or social pressure to the aforementioned 3 items (univaraite OR ranged from 1.85 to 5.86, p < 0.01) and to have given responses indicating perceived discrimination/social pressure to all 3 questions (59.7% versus 24.7%; univariate OR = 4.52; Table 3).

With regard to the 5 individual items related to perceived social support, Group I participants were less likely than Group V respondents to have provided responses indicating the availability of social support (univariate OR ranged from 0.26 to 0.64, p < 0.05) or to have given ≥ 4 affirmative responses (out of 5) to the availability of social support (8.4% versus 21.2%, univariate OR = 0.34, p < 0.001; Table 3).

### A multivariate summary model

The results showed that Group I respondents were more likely than Group V respondents to be not employed full-time (OR = 2.37), to have had sex with male sex workers in the last 12 months (OR = 3.44), to have recruited MSM sex partners via the internet in the last 12 months (OR = 7.62), to be inconsistent condom users during anal intercourse with MSM in the last 12 months (OR = 2.66), to self-report having contracted STD in the last 12 months (OR = 25.71), to be afraid of having others know about their sexual orientation (OR = 1.86), to perceive a fair amount/a great deal of discrimination against MSM in Hong Kong (OR = 2.91), and to feel discriminated against due to their sexual orientation (OR = 3.61). Group I respondents were less likely than Group V respondents to perceive moderate to very high susceptibility to contracting HIV in the future (OR = 0.56), to have multiple MSM sex partners in the last 12 months (OR = 0.25), and to have all or most of their friends being MSM (OR = 0.18; Table 4).

**Table 4:** A multivariate model predicting whether respondents are internet- or venue-recruited^#^

	Group I		Multivariate OR
		
	*(n)*	Row%	(95%CI)
Socio-demographics			
**Employment status**			
Employed full-time (n = 388)	*(118)*	30.4	1.00
Not employed full-time (n = 178)	*(108)*	60.7	2.37(1.46,3.86)***
			
HIV-related knowledge & perceptions			
**Self-perceived susceptibility of HIV infection in the future**			
Little/very little/not certain (n = 377)	*(170)*	45.1	1.00
Moderate to very high (n = 189)	*(56)*	29.6	0.56(0.33,0.96)*
			
Number & type of MSM sex partners in the last 12 months			
**Number of MSM sex partners**			
One (n = 177)	*(94)*	53.1	1.00
More than one (n = 389)	*(132)*	33.9	0.25(0.14,0.42)***
**Having sex with MSM acquaintances recruited online**			
No (n = 379)	*(110)*	29.0	1.00
Yes (n = 187)	*(116)*	62.0	7.62(4.40,13.20)***
**Having sex with male sex workers**			
No (n = 519)	*(197)*	38.0	1.00
Yes (n = 47)	*(29)*	61.7	3.44(1.53,7.70)**
			
Unprotected anal intercourse (UAI) in the last 12 months			
**Engaged in any MSM UAI**			
No UAI/no anal sex (n = 377)	*(129)*	34.2	1.00
Yes, UAI (n = 189)	*(97)*	51.3	2.66(1.62,4.37)***
			
Self-reported STD			
**Self-reported contraction of STD**			
No (n = 555)	*(216)*	38.9	1.00
Yes (n = 11)	*(10)*	90.9	25.71(1.99,331.57)*
			
Self-acceptance of MSM sexual orientation			
**Fear of others knowing their sexual orientation**			
No (n = 204)	*(48)*	23.5	1.00
Yes (n = 362)	*(178)*	49.2	1.86(1.09,3.17)*
			
Perceived MSM-related discrimination			
**Perceived discrimination against MSM in Hong Kong**			
Very little/little (n = 212)	*(41)*	19.3	1.00
A fair amount/a great deal (n = 354)	*(185)*	52.3	2.91(1.70,4.99)***
**Felt being discriminated against because of their sexual orientation**			
No (n = 211)	*(35)*	16.6	1.00
Yes (n = 355)	*(191)*	53.8	3.61(2.04,6.39)***
			
Perceived social support			
A few or none (n = 332)	*(174)*	52.4	1.00
All or most of them (n = 233)	*(52)*	22.3	0.18(0.11,0.30)***

Among all 566 respondents.

Group V: Venue-recruited MSM; Group I: Internet-recruited MSM.

# All univariately significant variables summarized in Tables 1, 2 and 3 are considered as candidate variables.

*p < 0.05; **p < 0.01; ***p < 0.001.

### Factors associated with UAI with MSM in the last 12 months among Group I and Group V respondents

The results showed that among Group I respondents, those whose best friends were supportive of their sexual orientation (OR = 1.94) and those having all or most friends being MSM (OR = 2.38) were more likely than others to have engaged in UAI with MSM in the last 12 months. Among Group V respondents, those who gave responses indicating perceived discrimination against MSM in all the 3 relevant items (OR = 2.33) and those whose best friends were supporting of their sexual orientation (OR = 1.97) were more likely than others to have engaged in UAI with MSM in the last 12 month, while the reverse was true for those aged 30 or above (OR = 0.54; Table 5).

**Table 5:** Factors associated with UAI in the last 12 months (*among those having had anal sex*) - Venue-recruited MSM (n = 272) & internet-recruited MSM (n = 182)

	Group V				Group I			
		
	UAI		Univariate	Multivariate OR	UAI		Univariate	Multivariate OR
	Row%	(n)	OR	(95%CI)	Row%	(n)	OR	(95%CI)
Socio-demographics								
**Age groups**								
18-29	37.5	*(72)*	1.00	1.00	54.3	*(69)*	1.00	---
≥30	25.0	*(20)*	0.56*	0.54(0.29,0.98)*	50.9	*(28)*	0.87	
**Number of items showing perceived discrimination against MSM (to the above 3 items)**								
0 - 2 discrimination responses	30.1	*(63)*	1.00	1.00	60.8	*(45)*	1.00	---
3 discrimination responses	46.0	*(29)*	1.98*	2.33(1.28,4.26)**	48.1	*(52)*	0.60	
**Best friends supporting their sexual orientation**								
No/unaware of one's sexual orientation	25.0	*(20)*	1.00	1.00	42.5	*(34)*	1.00	1.00
Yes	37.5	*(72)*	1.80*	1.97 (1.07, 3.62)*	61.8	*(63)*	2.19*	1.94(1.05,3.57)*
**Number of friends having MSM behaviors**								
A few or none	34.7	*(43)*	1.00	---	47.9	*(67)*	1.00	1.00
All or most of them	33.3	*(49)*	0.94		71.4	*(30)*	2.72**	2.38(1.11,5.11)*

Group V: Venue-recruited MSM; Group I: Internet-recruited MSM.

Multivariate OR: Odds ratio obtained from stepwise multivariate logistic regression using univariately significant variables as candidate variables.

Variable considered included all those listed in Tables 1, 2 and 3. For Group V (venue-recruited MSM), univariately significant variables included age group, number of items showing perceived discrimination against MSM, and having best friends supporting one's sexual orientation. For Group I (internet-recruited MSM), univariately significant variables included having best friends supporting one's sexual orientation, and number of friends having MSM behaviors. Only multivariately significant variables are summarized in Table 5.

*p < 0.05; **p < 0.01; ***p < 0.001.

--- univariately not significant (and was not considered in the multivariate model)

## Discussion

Consistent with the results of some previous studies [14,15,20], internet-recruited MSM were more likely than their venue-recruited counterparts to have engaged in UAI and commercial sex, and to report having contracted STD in the last 12 months. The level of perceived HIV risk was however lower among internet-recruited MSM than among MSM recruited from venues. Different recruitment methods may therefore provide different results.

Internet-recruited MSM were less likely than venue-recruited MSM to have multiple MSM sex partners. It is possible that venue-goers meet many potential sex partners face-to-face in bars and saunas etc. and may end up having sex with some of them eventually, whilst internet-based sex networks are often constructed in a virtual reality. Sexual networks are important platforms for both HIV transmission [24,25] and HIV interventions. Network-based interventions might be more feasible for venue-based respondents than for internet-based respondents. The problem of consuming psychoactive substances or alcohol prior to sexual intercourse was also more severe among venue-recruited MSM respondents than among internet-recruited respondents. Alcohol, drugs, and relevant peer pressure are more likely to prevail in venues. The findings of this study are consistent with those reported in other countries [14]. This suggests that venue-based campaigns should strengthen harm reduction of substance use and alcohol misuse.

Stating whether internet-based or venue-based respondents are at higher risk may oversimplify the issue. The two sampling methods may not be accessing a single population. Some but not all MSM can be accessed via both the internet and venues. Our data did not allow us to assess the degree of overlap. Our data, however, informed us that survey results of risk behaviors in MSM depend on the mode of data collection.

This study represents one of few attempts made to discern the socio-cultural profiles of venue-recruited and internet-recruited MSM. It can be seen that as compared to their counterparts recruited from venues, internet-recruited MSM respondents were less likely to disclose their sexual orientation to family members and to accept their own sexual orientation, and more likely to fear disclosing their sexual orientation to others, and to date/marry a woman in order to hide their sexual orientation. Implementation of face-to-face peer education may hence be more difficult and less feasible for internet-based MSM. Instead, internet-based empowerment efforts may be relevant for this group. Empowerment among MSM would increase condom use [26]. This study has also shown that only a minority of the participants, especially internet-based ones, had family or best friends who were supportive of their sexual orientation, possibly reflecting the severity of social stigma against MSM in Hong Kong.

Our findings indicated no significant between-group differences in service utilization rates. However, reservations and fear related to disclosing one's MSM identity might prevent some internet-recruited MSM from visiting gay venues. Since most MSM studies were based on venue-based sampling methods, the results of which were used to design programs targeting MSM in general, these programs may not be the most appropriate for internet-based MSM.

With respect to factors associated with UAI for venue-recruited and internet-recruited MSM respondents, perceived discrimination was significant for the former group but not the latter; the reverse was true for having some or all friends having MSM behaviors. Having best friends supporting one's MSM sexual orientation was significant for both groups. It is therefore seen that the two groups of MSM have both common and different factors associated with UAI. Social networks and social support may lead to both safer and riskier sexual behaviors [8,24]. Having best friends to support one's sexual orientation was associated with UAI in both groups; the norm among their peers however may not favor condom use. Furthermore, internet-recruited respondents were relatively lacking in social support and may therefore be more affected by peer influences. This may explain partially why the variable related to having more MSM friends was significant in internet-recruited MSM but not the venue-recruited sample. It is however less clear why perceived discrimination matters in the latter group but not in the former group. Further research is required.

The present study has some limitations. Firstly, convenience-sampling was used as random sampling was not feasible. Many published MSM studies have used similar recruitment methods [e.g., 8]. Secondly, data were self-reported and may be subject to reporting bias, though most sex behavior studies are also self-reported [e.g., 8,14,19,21]. Respondents were assured of strict anonymity and privacy of the interviews. Thirdly, we did not ask about HIV status of the individuals. HIV positive individuals may have higher levels of risk behavior as compared to their HIV negative counterparts. An alternate explanation of our results may hence be due to a higher proportion of HIV positive MSM using the internet to seek sex partners or for sero-sorting. We cannot test this hypothesis with our results and further research is warranted. However, the involved website does not have any special contents catering HIV positive MSM and sero-sorting is unpopular in Hong Kong. HIV positive MSM are also not deferred from seeking partners from gay venues. Therefore, the bias should not be too serious. Fourthly, stronger social desirability bias may occur among venue-recruited participants as compared to internet-recruited participants, who did not need to face an interviewer. Fifthly, it is possible that a higher proportion of internet-recruited participants were living with a regular partner, with whom unprotected anal intercourse is usually more common than with casual partners. There also was a possibility of self-selection bias in the internet-recruited respondents. Finally, biological markers were not collected in this study.

## Conclusions

The results of this study have implications on research survey methodology. They reinforce the claim that for HIV-related risk behavior studies, results are heavily dependent on the mode of data collection. Most of the published MSM studies are venue-based [e.g., 6,8] and their results should be interpreted with care. The relatively new respondent-driven sampling method (RDS) is meant to give probabilistic sampling estimates and has recently been applied to different MSM populations [e.g., 27]. Comparisons between venue-based, internet-based, and RDS survey results are warranted to understand potential impacts due to different sampling methods.

The study findings remind HIV workers that MSM are not a homogeneous group. According to the social marketing framework, segmentation is required for effective HIV prevention [28]. The "orientation-mix" of the segmented audience has to be sorted out for designing effective HIV intervention. Such does not seem to have been emphasized adequately in existing programs.

## **Competing interests**

The authors declare that they have no competing interests.

## **Authors' contributions**

JL is the principal investigator of the project. HYT performed the statistical analyses and implementation of the project. Both JL and HYT were involved in the design of the study, interpreted the results, and wrote the manuscript. Both authors read and approved the final manuscript.

## Pre-publication history

The pre-publication history for this paper can be accessed here:

http://www.biomedcentral.com/1471-2458/10/232/prepub

## References

[B1] Advisory Council on AIDS HIV/AIDS statistics in Hong Kong (updated 31 March 2008) ACA Newsfile 2008 15 27

[B2] Centers for Disease Control and Prevention (CDC) HIV prevalence among populations of men who have sex with men--Thailand, 2003 and 2005 MMWR Morb Mortal Wkly Rep 2006 55 31 844 848 16902394

[B3] Centers for Disease Control and Prevention (CDC) Trends in HIV/AIDS diagnoses among men who have sex with men--33 States, 2001-2006 MMWR Morb Mortal Wkly Rep 2008 57 25 681 686 18583954

[B4] Ma X Zhang Q He X Sun W Yue H Chen S Raymond HF Li Y Xu M Du H McFarland W Trends in prevalence of HIV, syphilis, hepatitis C, hepatitis B, and sexual risk behavior among men who have sex with men. Results of 3 consecutive respondent-driven sampling surveys in Beijing, 2004 through 2006 J Acquir Immune Defic Syndr 2007 45 581 587 10.1097/QAI.0b013e31811eadbc 17577125

[B5] State Council AIDS Working Committee Office UN Theme Group on AIDS in China A joint assessment of HIV/AIDS Prevention Treatment and Care in China (2007) 2007

[B6] Choi KH Han CS Hudes ES Kegeles S Unprotected sex and associated risk factors among young Asian and Pacific Islander men who have sex with men AIDS Educ Prev 2002 14 472 481 10.1521/aeap.14.8.472.24114 12512848

[B7] Dudley MG Rostosky SS Korfhage BA Zimmerman RS Correlates of high-risk sexual behavior among young men who have sex with men AIDS Educ Prev 2004 16 4 328 340 10.1521/aeap.16.4.328.40397 15342335

[B8] Jones KT Johnson WD Wheeler DP Gray P Foust E Gaiter J Nonsupportive peer norms and incarceration as HIV risk correlates for young black men who have sex with men AIDS Behav 2008 12 41 50 10.1007/s10461-007-9228-5 17436075

[B9] Mansergh G Naorat S Jommaroeng R Jenkins RA Stall R Jeeyapant S Phanuphak P Tappero JW van Griensven F Inconsistent condom use with steady and casual partners and associated factors among sexually-active men who have sex with men in Bangkok, Thailand AIDS Behav 2006 10 743 751 10.1007/s10461-006-9108-4 16715348

[B10] Bowen AM Horvath K Williams ML A randomized control trial of internet- delivered HIV prevention targeting rural MSM Health Educ Res 2007 22 120 127 10.1093/her/cyl057 16849391 PMC2639555

[B11] Fernández MI Perrino T Collazo JB Varga LM Marsh D Hernandez N Rehbein A Bowen GS Surfing new territory: club-drug use and risky sex among Hispanic men who have sex with men recruited on the Internet J Urban Health 2005 82 1 Suppl 1 i79 88 15738317 10.1093/jurban/jti027 PMC3456161

[B12] Hidaka Y Ichikawa S Koyano J Urao M Yasuo T Kimura H Ono-Kihara M Kihara M Substance use and sexual behaviours of Japanese men who have sex with men: a nationwide internet survey conducted in Japan BMC Public Health 2006 6 239 10.1186/1471-2458-6-239 17002800 PMC1599727

[B13] Lau JT Lau M Cheung A Tsui HY A randomized controlled study to evaluate the efficacy of an Internet-based intervention in reducing HIV risk behaviors among men who have sex with men in Hong Kong AIDS Care 2008 20 820 828 10.1080/09540120701694048 18608057

[B14] Elford J Bolding G Davis M Sherr L Hart G Web-based behavioral surveillance among men who have sex with men: a comparison of online and offline samples in London, UK J Acquir Immune Defic Syndr 2004 35 421 426 10.1097/00126334-200404010-00012 15097159

[B15] Evans AR Wiggins RD Mercer CH Bolding GJ Elford J Men who have sex with men in Great Britain: comparison of a self-selected internet sample with a national probability sample Sex Transm Infect 2007 83 200 205 10.1136/sti.2006.023283 17135330 PMC2659092

[B16] Rhodes SD DiClemente RJ Cecil H Hergenrather KC Yee LJ Risk among men who have sex with men in the United States: a comparison of an Internet sample and a conventional outreach sample AIDS Educ Prev 2002 14 41 50 10.1521/aeap.14.1.41.24334 11900109

[B17] Special Preventive Programme Centre for Health Protection Department of Health Factsheet on PRiSM 2006 2007

[B18] Li SW Zhang XY Li XX Wang MJ Li DL Ruan YH Zhang XX Shao YM Detection of recent HIV-1 infections among men who have sex with men in Beijing during 2005 - 2006 Chinese Medical Journal 2008 121 1105 1108 18706227

[B19] Lau JT Kim JH Lau M Tsui HY HIV related behaviours and attitudes among Chinese men who have sex with men in Hong Kong: a population based study Sex Transm Infect 2004 80 459 465 10.1136/sti.2003.008854 15572614 PMC1744932

[B20] Lau JT Kim JH Lau M Tsui HY Prevalence and risk behaviors of Chinese men who seek same-sex partners via the internet in Hong Kong AIDS Educ Prev 2003 15 516 528 10.1521/aeap.15.7.516.24046 14711165

[B21] Lau JT Kim JH Lau M Tsui HY Prevalence and risk behaviors of Hong Kong males who seek cross-border same-sex partners in mainland China Sex Transm Dis 2004 31 568 574 10.1097/01.olq.0000137905.94154.0a 15480120

[B22] Zhang B Zeng Y Xu H Li X Zhou S Liao L Study on 1389 men who have sex with men regarding their HIV high-risk behaviors and associated factors in mainland China in 2004 Chinese Journal of Epidemiology 2007 28 32 36 [Chinese] 17575928

[B23] Lau JTF Cai W Tsui HY Chen L Cheng JQ Psychosocial factors in association with condom use during commercial sex among migrant male sex workers living in Shenzhen, mainland China who serve cross-border Hong Kong male clients AIDS Behav 2009 13 5 939 48 10.1007/s10461-009-9591-5 19690951

[B24] Choi KH Ning Z Gregorich SE Pan QC The influence of social and sexual networks in the spread of HIV and syphilis among men who have sex with men in Shanghai, China J Acquir Immune Defic Syndr 2007 45 77 84 10.1097/QAI.0b013e3180415dd7 17325608

[B25] Ward H Prevention strategies for sexually transmitted infections: importance of sexual network structure and epidemic phase Sex Transm Infect 2007 83 Suppl 1 i43 49 10.1136/sti.2006.023598 17389716

[B26] Haque A Ahmed S Community based risks reduction approach among MSM: Bandhu Social Welfare Society: HIV/AIDS/STD prevention program. (Abstract no. WePeD4745) Proceedings of the 13th International Conference on AIDS, Durban, South Africa, July 9-14 2000

[B27] He Q Wang Y Li Y Zhang Y Lin P Yang F Fu X Li J Raymond HF Ling L McFarland W Accessing men who have sex with men through long-chain referral recruitment, Guangzhou, China AIDS Behav 2008 12 4 Suppl S93 96 10.1007/s10461-008-9388-y 18389358

[B28] Lamptey PR Price JE Social marketing sexually transmitted disease and HIV prevention: a consumer-centered approach to achieving behaviour change AIDS 1998 12 Suppl 2 S1 9 9792356

